# Satisfaction and Motivation of General Physicians toward Their Career

**DOI:** 10.5539/gjhs.v5n1p166

**Published:** 2012-11-19

**Authors:** Ameneh Barikani, Maryam Javadi, Aagil Mohammad, Barikani Firooze, Mojtaba Shahnazi

**Affiliations:** 1 Qazvin University of Medical Science, Qazvin, Iran; 2 General Practitioner, Qazvin, Iran; 3 Alborz Education Center, Tehran, Iran

**Keywords:** satisfaction, motivation, general physicians

## Abstract

**Background:**

Human resource in health system especially in developing countries has main role in health promotion. Therefore their satisfaction and motivation are the key points in developing health system.

**Objective:**

To determine the motivation and satisfaction of general physicians (GP) towards their career.

**Methods:**

Using random sampling, 150 physicians were selected from comprehension commercial database list. Data were collected using a self-administered questionnaire that consisted of three sections; first demographic data, second work satisfaction and third questions toward biologic, dependent and growth motivation. Data were analyzed by SPSS version 16 with P<0.05.

**Results:**

From participants 64.7% of physicians were in age between 30-40 years and 27.3% were men. Only 5.3% of physicians who were employed for over 10 years were satisfied from their career. Satisfaction of career among female and male physicians was 8% and 24% respectively. The item of job safety sensation in biologic motivation had maximum scale (4.1±0.89). In dependent and growth motivations, value success sensation in job (4+-0.88) and make new skills and knowledge (4+-0.67) had maximum scale of mean. Relation of growth motivators with age (P<0.01), postgraduate duration (P<0.005) was significant. Dependent motivators had significant relation with age (P<0.04), postgraduate duration (P<0.01) and employment duration (P<0.002). Biological motivators had significant relation with sex (P<0.4) and satisfaction of work hours (P<0.007). Correlation of biological (r=0.44, P<0.001) and growth (r=0.7, P<0.001) motivators was significant.

**Conclusion:**

Growth motivators score had higher ranking than other motivators. However, biological motivators especially job security and finance were also important and must be noticed from decision makers.

## 1. Introduction

Motivation is a process of arousing and sustaining goal-directed behavior induced by the expectation of satisfying individual needs. An employee’s performance typically is influenced by motivation, ability and the work environment. Knowing how and why to motivate employees is an important managerial skill.

Change and the sustainability of change, depend on human qualities rather than on the quality of equipment or the quantity of money available. Improvements in health services require a motivated workforce to implement them and to ensure their survival during difficult times (Maclachlan & Carr, 1993; Freiderike, 2009). The theories of motivation are generally categorized into two groups: content theories and process theories. Content theories focus primary on individual needs, attempting to explain the factors within a person that energize and stop behavior. They address the question “what factors motivate people?

Examples of content theories are Maslows hierarchy of needs theory (Scott- Myers, 1964). Alderfer’s ERG theory (Alderfer, 1972), Herzberg’s two-factor theory (Herzberg et al., 1959) and Mccelland’s needs theory (McClelland, 1961).

Process theories focus on why and how of motivation investigating formally into the thinking process through which people choose one action versus another in the workplace (Ruthankoon & Ogunlana, 2003).

Motivation has been a popular research topic for over 50 years. Performance and productivity of workers should still be important topics for management research in future, especially for knowledge workers who are valuable assets to institutions (Drucker, 1973). Motivation is a process that results from the dynamic interactions between individuals, their work environment and community or society (Franco et al. 2004; Bennett & Kanfer, 2002).

In developing countries because of accessibility of health care is low, motivation of human resources is very important. In a study in Pakistan motivating factors were intrinsic and social cultural factors like serving people, respect and career growth (Malik et al., 2010).

The word motivation was derived from Latin term movers which means to move Definitions of motivation tend to center around now to provide something to a person to drive him (or her) to do something (Ruthankoon & Ogunlana, 2003).

Productivity is defined as the efficient and effective use of resources with minimum waste and effort to achieve income. We live in a world that has limited resources. The health care leaders of health care organization are increasingly interested in ways to attract, retain and gain commitment from their employees. This interest is created in part because high turnover rates and the lack of commitment negatively affect the provision of care and the bottom live in their organizations. In a quality and cost conscious health care environment, health care managers need to find solutions for these difficult issues, solutions that are effective, efficient and sustainable.

According to World Health Organization(WHO) there is a worldwide estimation about shortage of 4.3 million health workers, primarily concentrated in south Asia followed by Africa (Malik et al., 2010; WHO, 2006). The burden of disease in these two regions is high. In other words Sub Saharan Africa and Southeast Asia together have 53% of Global Burden of Disease, but 15% of Worlds health care workforce (Raman, 2008; Malik et al., 2010). In Iran GP have responsibilities of management of health system and primary care.

The general practitioners in Iran currently face different changes in economical and job safety. GP employed in the area that are under constant pressure to do more with less, that lead to high levels of employee dissatisfaction. Physician satisfaction is a critical aspect of quality health care, allowing the development of a motivated workforce and committed to improving patient outcomes.

The study of motivation in developing countries, especially in Iran was low, therefore this study with aim assessment of motivation and satisfaction in GP was done.

## 2. Material and Methods

With a cross sectional study a random sample of 150 general physicians was drawn from a comprehension commercial database listing all 400 physicians actively practicing in Qazvin city a province of Iran Data were collected in 2008, with using a self-administered questionnaire that contain questions such as age, sex, duration of graduate and employee, income, second was job satisfactions of physical environment, opportunity for use of skills, income and work time and third part, 25 questions toward motivation base of Alderfer ERG (Existence, Relatedness, and Growth) motivation theory. Clayton Alderfer derived the ERG theory, which is an extension of Maslow’s hierarchy of needs. Alderfer classified the needs into the categories: Existence, relatedness, and growth. According to Alderfer, the needs aren’t in any order and any desire to fulfill, a need can be activated at any point in time. These results showed that, the lower level needs not requiring to be satisfied in order to satisfy a higher level needs. Alderfer’s ERG theory can actually be utilized as a frustration-regression principle where an already satisfied lower level need can be “re-activated” when confronted with the impossibility of satisfying a higher level one.

Existence, related to person’s physical needs such as food, clothing and shelter. Relatedness, relates to a person’s interpersonal needs within his personal as well as professional settings. Growth relates to a person’s needs of personal development (Alderfer, 1972). Internal consistency of questionnaire with Cronbachs Alpha was 86%. Data were analyzed with Chi^2^ test and spearman correlation by SPSS version16 with P<0.05.

## 3. Results

From total of participants, 27.3% were men. Postgraduate time was 5-10 year in 42.7% (Table 1).

**Table 1:** Characteristics of physicians

	NO	%
Age (year)		
- <30	32	21.3
- 30-40	97	64.7
- >40	21	14
Postgraduate time (year)		
- <5	36	24
- 5-10	64	42.7
- >10	50	33.3
Employment time (year)		
- <5	37	24.7
- 5-10	66	44
- >10	47	31.3
Sex		
- men	41	27.3
- women	109	64.7

26.7% of them had high satisfaction toward their career (Table 2).

**Table 2:** Satisfaction of physicians toward their career

	Yes	SOMEDEAL	NO	P
Income				
- high	6	0.7	0 4	0.000
- moderate	11.3	12.7	24.7
- low	14.7	26	
Hours of work				
- very	5.3	1.3	0.7	0.000
- moderate	16	10.7	1.3	
- few	10.7	27.3	26.7	
Knowledge about profession				
- yes	7.3	8	1.3	0.000
-SOMEDEAL	15.3	20.7	6.7	
- no	9.3	10.7	20.7	
Employment time				
- <5	13.3	8.7	2.7	0.003
- 5-10	13.3	15.3	16.3
- >10	5.3	15.3	10.7
Postgraduate time				
- <5	13.3	8	2.7	0.006
- 5-10	11.3	16	15.3
- >10	7.3	15.3	10.7
Sex				
- female	8	16	3.3	0.005
- male	24	23.3	25.3	
Age (year)				
- <30	12.7	6.7	2	0.001
- 30-40	14	28.7	22	
- >40	5.3	4	4.7	
Physical environment				
- Good	6	1.3	0.7	0.000
- not bad	18	14.7	6	
- bad	8	23.3	22	
Opportunity for use of skill				
- yes	6	2	3.3	
- accessional	14.7	25.3	6.7	
- no	11.3	12	15.7	

Job safety sensation in biological motivation with score of 4.1+-0.89, value success sensation in job in dependent motivation with score of 4+-0.88 and new skills and knowledge in growth motivation with score of 4 +-0.67 had highest score (Table 3)

**Table 3:** Mean score of motivation in physicians

	Mean	SD
Biological motivation		
- job safety sensation	4.1	0.89
- merit available	3.7	0.99
- effect of job on private life	3.7	1.01
- income	3.9	0.89
- convenience availability appropriate	3.8	0.77
- workplace room appropriate	3.9	0.75
Dependent motivation		
- value success sensation in job	4	0.88
- high level job and population notice	3.7	0.81
- to put in practice	3.6	0.8
- independence judgment	3.6	1.05
- good relationship with others	4	0.66
- to draw patient confidence	4.1	0.75
- team work possibility	3.3	0.98
- good relationship with colleagues	3.5	0.93
- Belonging sensation to population	3.7	0.93
- patient appreciation	3.5	0.89
- self-respect sensation	3.3	1.7
Growth motivation		
- opportunity for self-improvement	3.9	0.83
- opportunity for receive continual education	3.7	0.83
- receive colleagues experiment	3.4	0.8
- new skills and knowledge	4	0.67

In this study score of biological motivators in men was higher than women (P=0.04).

**Table 4:** Mean score of three motivations with character of physicians

	Biological	Dependent	Growth
Age (year)			
- <30	4±0.49	3.8±0.31	4±0.29
- 30-40	3.7±0.61	3.6±0.61	3.8±0.53
- >40	3.8±0.66	3.9±0.4	4.1±0.41
P	0.1	0.04	0.01
Sex			
- female	3.6±0.6	3.6±0.6	3.8±0.5
- male	3.9±0.58	3.7±0.5	3.9±0.48
P	0.04	0.1	0.1
Postgraduate duration (year)			
- <5	3.9±0.56	3.8±0.31	4±0.33
- 5-10	3.7±0.66	3.8±0.52	3.8±0.55
- >10	3.8±0.53	3.7±0.53	3.9±0.48
P	0.2	0.01	0.05
Employment duration (year)			
- <5	3.9±0.56	3.8±0.4	4±0.37
- 5-10	3.8±0.66	3.8±0.5	3.6±0.54
- >10	3.7±0.52	3.7±0.53	3.9±0.48
P	0.3	0.02	0.1
Satisfaction of income			
- yes	3.5±0.47	4±0.43	4.1±0.42
- no	3.9±0.56	3.6±0.55	3.9±0.46
P	0.5	0.2	0.7
Satisfaction of work hours			
- yes	3.9±0.57	4.2±0.58	4.3±0.44
- no	3.7±0.62	3.6±0.52	3.8±0.51
P	0.007	0.09	0.2

High level needs in physicians very important in 38.7%.

**Table 5:** Percent of motivation important in view of physicians (n=150)

	Very	Moderate	Low
Biological	29.3	56.7	14
Dependent	29.3	56.7	14
Growth	48	48.7	3.3

## 4. Discussions

Human resource in health system has central role in improving health, motivation and satisfaction. In this study aspects of biological motivation was important. Biological need was one of the basic needs that is neglected but could effect on motivation. Among different aspect of biological motivators, job security is most important. If physicians have job security, then other aspects of biological motivators will be influenced by job security. Unemployment in medical graduates in many studies in Iran was 12.2% (Mirkamali, 1990; Dehghani, 2007). Most of physicians especially young physicians have concern about future career and job security. According to long duration of education in this profession, physicians have high expectations and job security is the minimum request of them.

Organizational factors particularly in current job settings could be the most de motivator, a finding that echoed in other studies (Reid, 2004; Chikanda, 2005; Ssengooba et al., 2007; Malik et al., 2010). Although money is not only motivator, but is important along with others motivators. If physicians have financial problems may have a tendency to un medical activities in addition to decrease motivation. In a study in Iran, 15% of GP work in un medical profession and 5% were abroad. In this study different aspects of dependent motivator were important, among these motivators, value success sensation was most important. This sensation can guide individuals to value objects. In other word disappointment sensation and unimprovement in the job is a strong motivator. Certain intrinsic and socio-cultural factors such as respecting people and opportunities for career growth are important motivators. Recognition by employers and communities and draw their net ice and good relationship with them and colleagues was important (Franco et al., 2004; Agyepong et al., 2004; Dieleman, Toonen, Toure & Martineau, 2006; Manongi, Marchant & Bygbjerg, 2006; Ssengooba et al., 2007).

In compare growth motivators was more important than others. Individuals that practice as physician have high level expectations. In related their job, as if biological and dependent motivators have intermediate role. Decision makers must be notice this fact for improving function of health system. According to Alderfer theory an individuals can move from low levels of needs to high on the condition that they struggle to meet their needs, in this way decision makers must notice to high level needs of their employers (Moghimi, 2006). In a study in Vietnam, most motivators were managers and colleagues gratitude, job security, sufficient income and education (Dieleman, 2003). Lack of opportunities for higher specialization is one of important de motivator as seen in other studies (Malik et al., 2010). Tendency to specialization could be refer to financial and job security problems. Tend to continue of education after duration of general practitioner (GP) in Iranian physicians is very high (Nejat et al., 2007; Buddeberg-Fischer et al., 2006; Lambert et al., 2006). This fact must be noticed by decision makers. Satisfaction of hours of work was 7.3% in our study, this results was not similar to Al-Eisa study (Ibrahim, Manal & Huda, 2005; Catherine M Joyce et al., 2011). Barnes said that hours of work is one factor that can affect internal satisfaction, directly (Barnes, 1998). Dwell believed that long duration hours of work is one of unsatisfaction factors (Dowell, Hamilton, & McLeod, 2000). In our study 16.7% 0f physicians had sufficient knowledge about their profession.

These problems refer to insufficient information toward medical profession at the time of entering to university to start medical education. These students have not any knowledge about their responsibilities and problem of their profession that was not similar to Al-Eisa study (Ibrahim, Manal & Huda, 2005). In our study satisfaction in young physicians was higher than old, that was not similar to other studies (Ibrahim, Manal & Huda, 2005; Haas et al., 1998). Young physicians often have not enough information about their responsibility and problems that may be faced with it. Our results showed that satisfaction of male physicians was higher than female, that was not similar to other studies (Okerlund, Jacson, & Parsons, 1994; McMurray et al., 2000; Ibrahim, Manal & Huda, 2005) and similar to Frank and Malik study (Frank, McMurray, Linzer, & Elon, 1999; Pas et al., 2008; Malik et al., 2010). Household responsibilities and restriction for working in anywhere and region can affect their satisfaction and motivation. Our study showed that employment time have affect on satisfaction and follow motivation. Physicians that their employment time was higher than 10 years were more satisfied, that was not similar to Al-Eisa study (Ibrahim et al. 2005, Manal & Huda, 2005). Physicians soon after postgraduate have not job stability, because want to participate in specialty exam or spend their legal duty in health system (Tarh). In our study relation of growth motivation with satisfaction was significant (P<0.01) similar to Okerlund study (Okerlund, Jacson & Parsons, 1994). Also high level motivation in workplace can drive individuals’ attention from low levels needs to high but managers must notice to three level of motivation, because significant relation was seen between different levels of motivation.

Motivation does not remain static and is dependent on many continually changing factors in a community like Iran that younger physicians faced to economic problems.

This study was first of its hind in the region to investigate physician’s motivation. The results of this study showed that if biological motivators were important, but most of physicians have growth needs that must be noticed by decision makers.
